# Simultaneously enhanced optical, electrical, and mechanical properties of highly stretchable transparent silver nanowire electrodes using organic surface modifier

**DOI:** 10.1080/14686996.2019.1568750

**Published:** 2019-02-18

**Authors:** Siti Aisyah Nurmaulia Entifar, Joo Won Han, Dong Jin Lee, Zeno Rizqi Ramadhan, Juhee Hong, Moon Hee Kang, Soyeon Kim, Dongchan Lim, Changhun Yun, Yong Hyun Kim

**Affiliations:** a Department of Display Engineering, Pukyong National University, Busan, Republic of Korea; b Department of Electrical Energy Engineering, Keimyung University, Daegu, Republic of Korea; c Surface Technology Division, Korea Institute of Materials Science (KIMS), Changwon, Republic of Korea; d Center for Nano-Photonics Convergence Technology, Korea Institute of Industrial Technology (KITECH), Gwangju, Republic of Korea

**Keywords:** Stretchable electronics, transparent electrodes, silver nanowires, PEDOT:PSS, transparent heaters, 40 Optical, magnetic and electronic device materials, 102 Porous / Nanoporous / Nanostructured materials, 212 Surface and interfaces, 204 Optics / Optical applications

## Abstract

We report on a new surface modifier which simultaneously improves electrical, optical, and mechanical properties of silver nanowire-based stretchable transparent electrodes. The transparent electrodes treated with 11-aminoundecanoic acid achieve a low sheet resistance of 26.0 ohm/sq and a high transmittance of 90% with an excellent stretchability. These improvements are attributed to the effective formation of a strong chemical bond between silver nanowire networks and elastomeric substrates by 11-aminoundecanoic acid treatment. The resistance change of the optimized silver nanowire/poly(3,4-ethylenedioxythiophene):poly(styrenesulfonate) (PEDOT:PSS) thin-films is only about 10% when the film is stretched by 120%. In addition, the chemical stability of stretchable silver nanowire films is significantly improved by the introduction of conductive PEDOT:PSS overcoat film. The optimized electrodes are utilized as high-performance stretchable transparent heaters, successfully illustrating its feasibility for future wearable electronics.

## Graphical abstract

**Figure:**
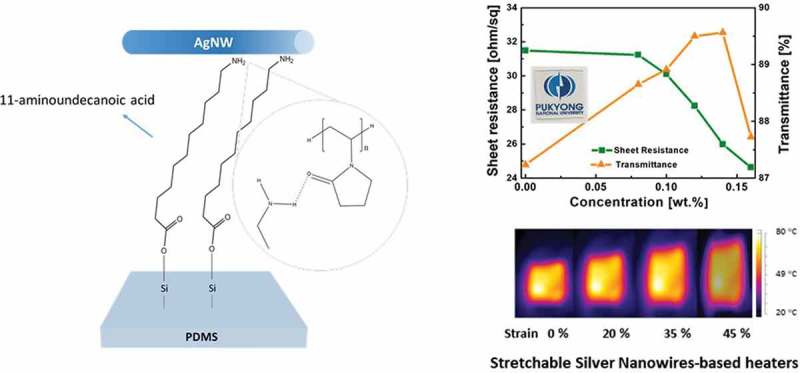

## 1. Introduction

High-performance stretchable transparent conductive electrodes (TCEs) are of great necessity for the development of stretchable optoelectronics, which can be integrated in devices with new form factors, such as textiles, skin-based devices, and wearable devices [1,2]. Indium tin oxide (ITO) is the most generally used TCE in optoelectronics such as organic light-emitting diodes and various solar cells owing to its high-optical transmittance and high-electrical conductivity. However, the application of ITO is strongly limited in stretchable electronics due to its high costs, inherent brittleness, and high-temperature manufacturing process [3]. Graphenes [4,5], carbon nanotubes [6,7], conductive polymers [8–11], metal grid [12–14], and metal nanowires [15–17] have thus been widely investigated as alternative TCEs to replace ITO. However, these alternative TCEs still suffer from a number of challenges including high-electrical resistance or poor stretchability on stretchable substrates which limit their application in wearable electronic devices. Teo et al. reported the conductive PEDOT:PSS on polydimethylsiloxane (PDMS) where the sheet resistance increased by 2.3 times at 50% strain [18]. Hu et al. demonstrated the sheet resistance of silver nanowires (AgNWs) embedded in a crosslinked poly(acrylate) increased by 2.3 times at 50% strain [19]. AgNWs on PDMS reported by Liu et al. showed the increase of sheet resistance by 1.9 times at 100% strain [20]. The sheet resistance of copper nanowires on polyurethane substrates demonstrated by Hu et al. increased by 6.4 times at 50% strain [21]. AgNWs are one of the promising stretchable TCEs because it offers high conductivity, high transmittance, good mechanical performances, and easy solution processability [22]. The AgNW networks achieve a sheet resistance and a transmittance comparable to those of ITO. With these excellent electrical and optical properties of AgNWs, there have been many studies for applications of AgNW-based TCEs in organic solar cells, organic light-emitting diodes, touch panels, transistors, and stretchable electronic devices [22–24].

The approaches in achieving stretchable electrodes can be described as follows: (i) deposit conductive materials with new structural layouts such as wavy, meshed, percolated, or buckled geometries on an elastomeric substrate or (ii) deposit conductive materials in surface-modified elastomeric substrates [24,25]. The AgNW networks as well as other TCEs on highly hydrophobic surfaces of elastomeric substrates such as PDMS typically suffer from poor adhesiveness to substrates [25–27]. Moreover, the TCE films can be easily peeled-off from the elastomeric substrate when small mechanical forces are applied. However, there have been a few studies especially for AgNWs to overcome such issue, up to now. Exposure to oxygen plasma or introduction of aqueous hydrochloric acid are ways to realize defined surface properties of elastomeric substrate [17,25,26,28].

In this study, we report simultaneously improved electrical, optical, and mechanical properties of AgNW-based stretchable TCEs by incorporating 11-aminoundecanoic acid (11-AA) as the novel surface modifier processed by simple spin-coating. The TCEs treated with 11-AA achieve a low sheet resistance of 26.0 ohm/sq and a high transmittance of 90%. This high performance is attributed to the effective hydrogen and covalent bonding formed between AgNW networks and highly hydrophobic PDMS substrates with 11-AA. Furthermore, we develop 11-AA treated AgNWs/conductive poly(3,4-ethylenedioxythiophene):poly(styrenesulfonate) (PEDOT:PSS) composites, resulting in the enhanced chemical stability and stretchability of stretchable thin-films. The change of resistance for AgNW/PEDOT:PSS hybrid electrodes optimized with 11-AA is only 11% under a strain of 120%. With the use of 11-AA, the electrical properties are retained under high tensile strain and repeated stretching conditions. In addition, the optimized composite TCEs are utilized to transparent heaters, showing much enhanced Joule heating performance. The stretchable devices exhibit superior elastic behaviors, which can be stretched, bent, and twisted without performance degradation. The AgNWs optimized with 11-AA treatment and overcoating PEDOT:PSS can provide a new path toward high-performance stretchable transparent electrodes for robust stretchable electronics.

## 2. Experimental details

The stretchable transparent electrodes were prepared in air ambient at a constant relative humidity of around 40% and room temperature condition. As stretchable substrates, PDMS solutions (Sylgard 184, Dow Corning, USA) were spin-coated on to polyethylene terephthalate (PET) at 300 rpm for 15 s, and were subsequently annealed at 120 °C for 40 min. The prepared PDMS substrates were pretreated by oxygen plasma for 15 min. For the surface modification of PDMS, 11-AA solutions (Sigma Aldrich, USA) with a concentration of 0.08–0.16 wt.% in deionized water were spin-coated onto plasma-treated PDMS samples, and subsequently annealed at 100 °C for 10 min. The aqueous 0.5 wt.% AgNW aqueous dispersion (Nanopixys, Korea) was spin-coated onto PDMS substrates at 3000 rpm and was baked on the hot plate at 100 °C for 10 min. The average length and diameter of the AgNWs were about 25 μm and 32 nm, respectively. For the fabrication of AgNW/PEDOT:PSS composites, PEDOT:PSS (Clevios FET, Heraeus, Germany) was spin-coated on the AgNW films at 8000 rpm for 30 s and annealed at 120 °C for 10 min. The dried samples on the PDMS were peeled-off from the PET supporting substrates and cut into the required sample size. The photographs of samples at various stages are shown in the Supplementary Material. For the fabrication of transparent heaters, aluminum tapes were attached on both edges of the AgNW or AgNW/PEDOT:PSS composites electrodes, which were wired to the power supply.

The sheet resistance was examined by van der Pauw method using a source-measure unit system (Keithley 2401, USA) at room temperature. The electrical contacts were made by coating a liquid gallium–indium eutectic alloy, which provides a good adhesion on the PDMS substrate, on the four corners of each film. The transmittance was measured by UV−vis spectrophotometer (Optizen pop, MECASYS, Korea). The transmittance values of films are specified at a wavelength of 550 nm and do not include the substrate. The transmittance of PDMS is about 94% at a wavelength of 550 nm. The relative resistance under tensile strains was measured by a Keithley sourcemeter with a custom-made motion stage. For chemical stability tests, the TCEs were immersed in the solvent bath containing either ethanol or deionized water. DC voltage was applied to both sides of the electrodes by a Keithley 2401 sourcemeter to induce Joule heating. Thermal properties and images were obtained using an infrared (IR) camera (TiS45, Fluke, USA).

## 3. Results and discussion

**Figure 1.**
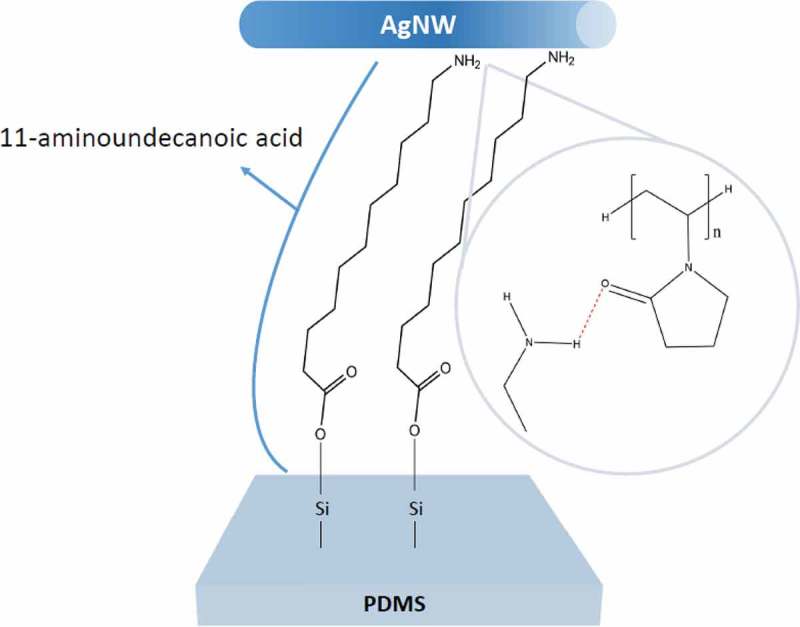
Schematic illustration of the formation of chemical bonds with 11-AA between the AgNW and the PDMS substrate. The primary amine (–NH_2_) group in 11-AA forms a hydrogen bond to PVP-capped AgNWs. The carboxyl group (COOH) of 11-AA forms a covalent bond with PDMS substrates.

The AgNWs are deposited on PDMS which has a high transmittance, high elasticity, and outstanding biocompatibility. Figure 1 shows the schematic mechanism of the enhanced bonding properties using 11-AA, which has 11 carbon atom chains with a carboxyl group and an amine group at each end. Due to the functional groups, 11-AA can be effectively coated onto nanoparticles by the layer-by-layer self-assembly [29,30]. However, 11-AA has not been used as a surface modifier for improving the adhesion of the nanowire and elastomeric substrates. Due to the weak van der Waals forces between AgNWs and the underlying PDMS substrate, a surface treatment step is required in order to produce strong, conformal bonds between the AgNW networks and the surfaces of PDMS. The plasma treatment for PDMS creates silanol groups (–OH) at the surface [17,26]. Additionally, we introduce 11-AA on plasma-treated PDMS substrates. The functional group of primary amine (–NH_2_) in 11-AA forms a hydrogen bond to polyvinylpyrrolidone (PVP)-capped AgNWs. Furthermore, the carboxyl group (COOH) of 11-AA forms a strong covalent bond with PDMS substrates. Covalent bonds are several orders of magnitude stronger than van der Waals forces [17]. In addition, the hydrogen bond formed between AgNWs and 11-AA provides a strong bonding force. Thus, the resulting covalent and hydrogen bonds formed by 11-AA can significantly improve the adhesion between nanowire and elastomeric substrate.

**Figure 2.**
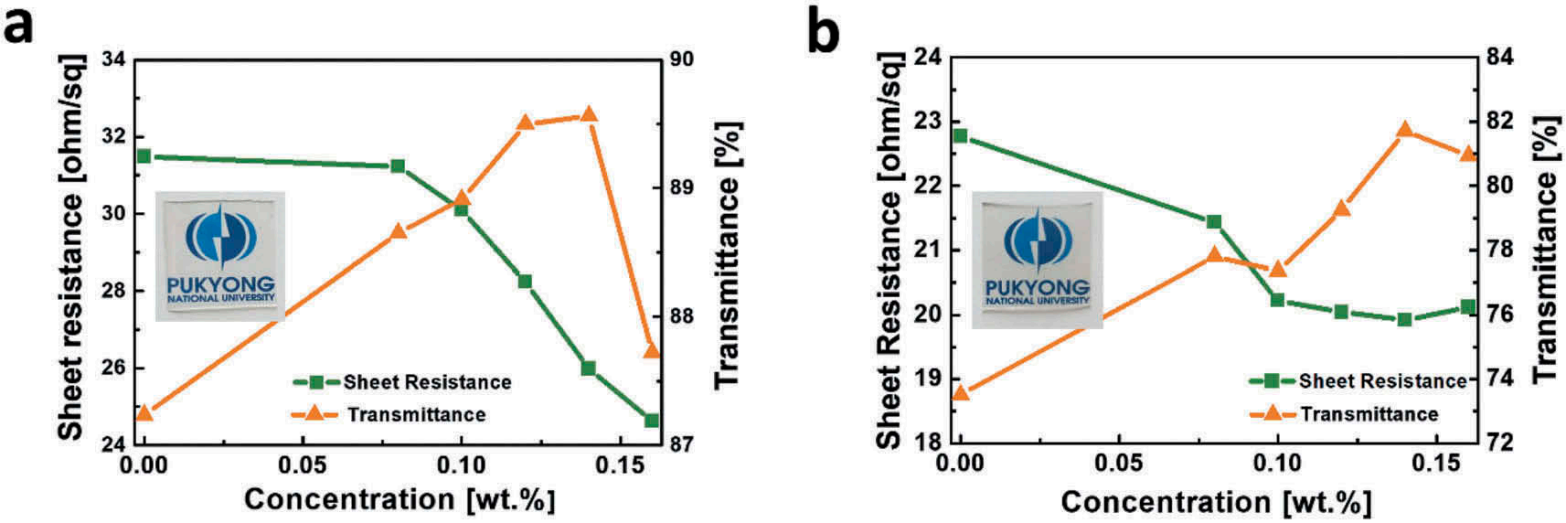
Sheet resistance and transmittance at 550 nm of (a) AgNW and (b) c-AgNW treated with various concentration of 11-AA. The best performing AgNW and c-AgNW are observed at an 11-AA concentration of 0.14 wt.%. The corresponding films are presented in the insets.

Figure 2(a) shows the behavior of the sheet resistance and the transmittance of AgNW networks as a function of the concentration of 11-AA in deionized water. The sheet resistance of reference sample without 11-AA reveals a sheet resistance of 31.5 ohm/sq with a transmittance of 87%. As the concentration of 11-AA increases, the sheet resistance decreases while the transmittance increases. The best performing AgNW network shows a sheet resistance of 26.0 ohm/sq and transmittance of 90% at an 11-AA concentration of 0.14 wt.%. Such well-bonded AgNW network by 11-AA shows simultaneously enhanced electrical and optical properties. At a higher 11-AA concentration of 0.16 wt.%, the transmittance drops to 88% while sheet resistance further decreases up to 24.6 ohm/sq. The well-bonded nanowires to the PDMS substrate significantly improve the wire-wire contacts, resulting in the reduced sheet resistance. In contrast, loose wires on PDMS substrates without 11-AA treatment inevitably weaken wire-wire contact points. The improved transmittance of AgNWs with 11-AA treatment is attributed to the lowered haziness with well-distributed nanowires.

We also investigate the electrical and optical properties of combined AgNW-conductive PEDOT:PSS composites (c-AgNW) as shown in Figure 2(b). The overcoated PEDOT:PSS enhances the adhesion properties and chemical stability of the AgNW-based films as well as thermal properties in AgNW-based stretchable devices as described below. Owing to the conducting PEDOT:PSS coatings, the transmittance of c-AgNWs decreases, as compared to that of AgNW networks, with decreasing sheet resistance. The best performing c-AgNW film shows a sheet resistance of 19.9 ohm/sq with transmittance of 82% at an 11-AA concentration of 0.14 wt.%. These values, both for AgNWs and c-AgNWs, are promising for applications in optoelectronic devices.

**Figure 3.**
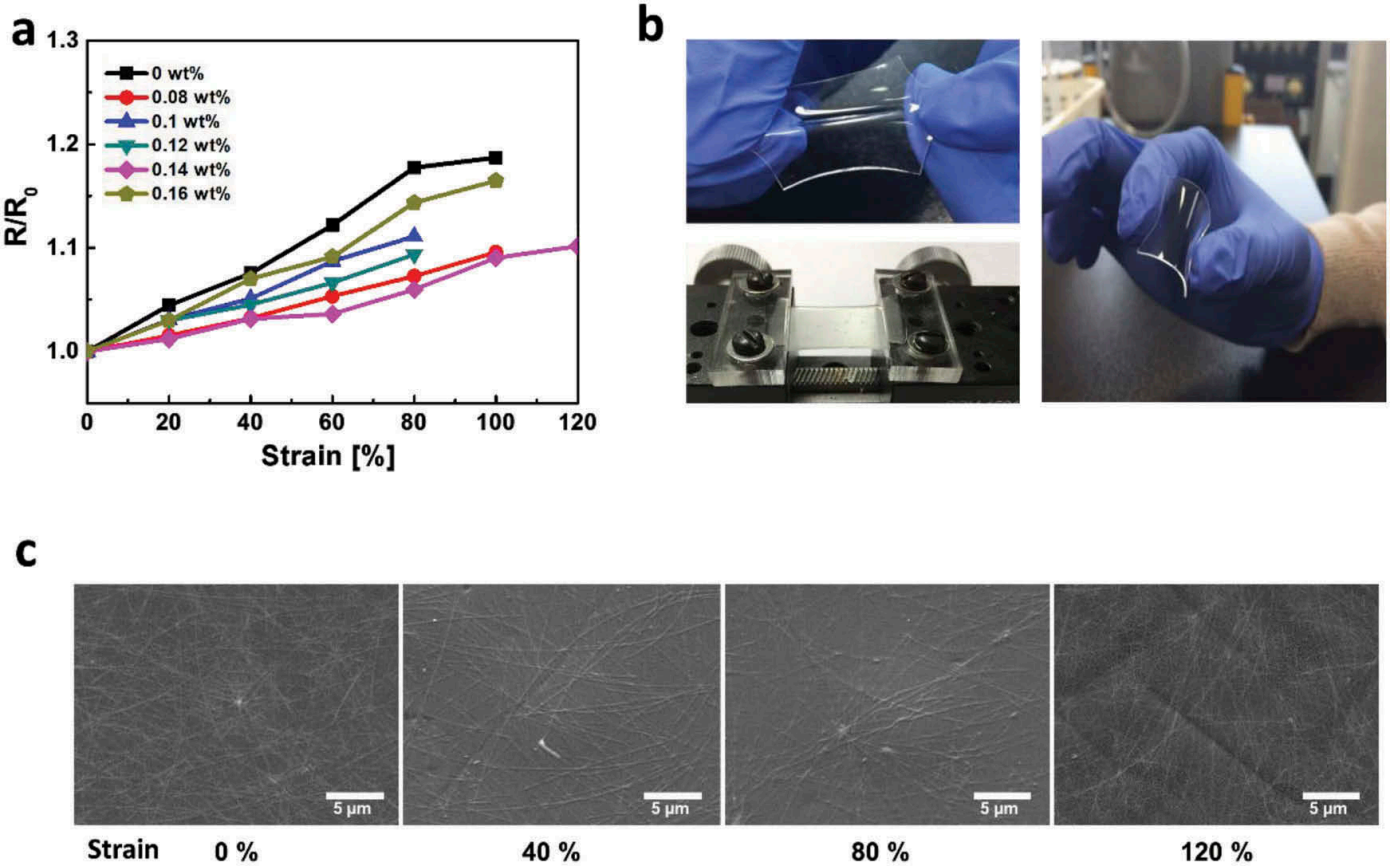
(a) Resistance of c-AgNWs treated with various concentration of 11-AA as a function of tensile strain. (b) Photographs of c-AgNW under stretching and bending conditions. (c) Scanning electron microscopy images of stretched c-AgNW treated with 0.14 wt.% of AA under various tensile strains. The c-AgNWs are stretched up to 120%, resulting in the *R*/*R*_0_ increase by only 1.1-fold.

Figure 3(a) shows the relative changes of the resistance (*R*/*R*_0_, where *R* is the resistance under stretched condition, *R*_0_ is the initial resistance without strain) for c-AgNWs with respect to the concentration of 11-AA under the induced tensile strain. The resistance of films increases along with the applied strain. The c-AgNWs with an 11-AA concentration of 0.14 wt.% are stretched up to 120%, resulting in the *R*/*R*_0_ increase by only 1.1-fold. The 11-AA introduced-c-AgNW networks reveal a remarkably suppressed resistance increase when the concentration of 11-AA increases. This suggests that 11-AA improves bonding between AgNWs and PDMS substrates and substantially suppresses the disconnection of wires under strains so that conducting pathways are retained. This superb stretchability using 11-AA is favorable for achieving high-performance stretchable electronics with minimal electrical loss. Figure 3(b) presents photographs of c-AgNW under stretching and bending conditions. The resistance increase of stretchable electrodes is caused by microscopic cracks together with the disconnection of nanowires. The crack of c-AgNW films treated with 11-AA (0.14 wt.%) is observed at the strain of 120%, which leads to break of nanowire junctions and deteriorates the conductivity of stretchable electrodes.

**Figure 4.**
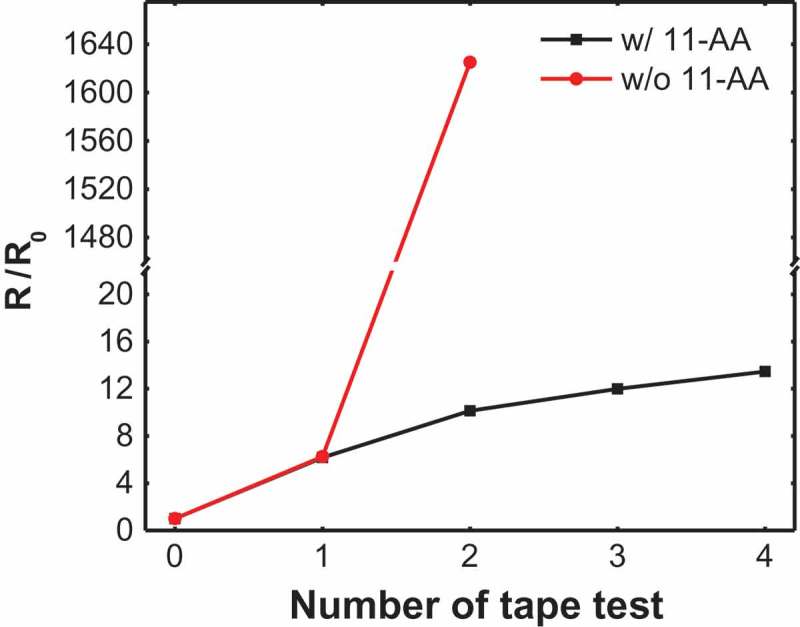
Changes of resistance for AgNWs with and without 11-AA treatment as a function of the number of tape test. The 11-AA treated AgNWs exhibit highly robust characteristics with a limited resistance change.

To investigate the adhesion properties between AgNWs and PDMS substrates, we perform the tape attach-release test for the films where the adhesive tapes are attached on top of the films and then peeled-off. Figure 4 shows the relative resistances of AgNW films with and without 11-AA treatment. The resistance of the untreated AgNWs dramatically increases over a few cycles of tape test, indicating the poor adhesion of AgNWs to the PDMS substrates. In contrast, the 11-AA treated AgNWs exhibit highly robust characteristics with a limited resistance change. These results confirm the strong enhancement of adhesion properties of AgNW/PDMS films by the 11-AA treatment.

**Figure 5.**
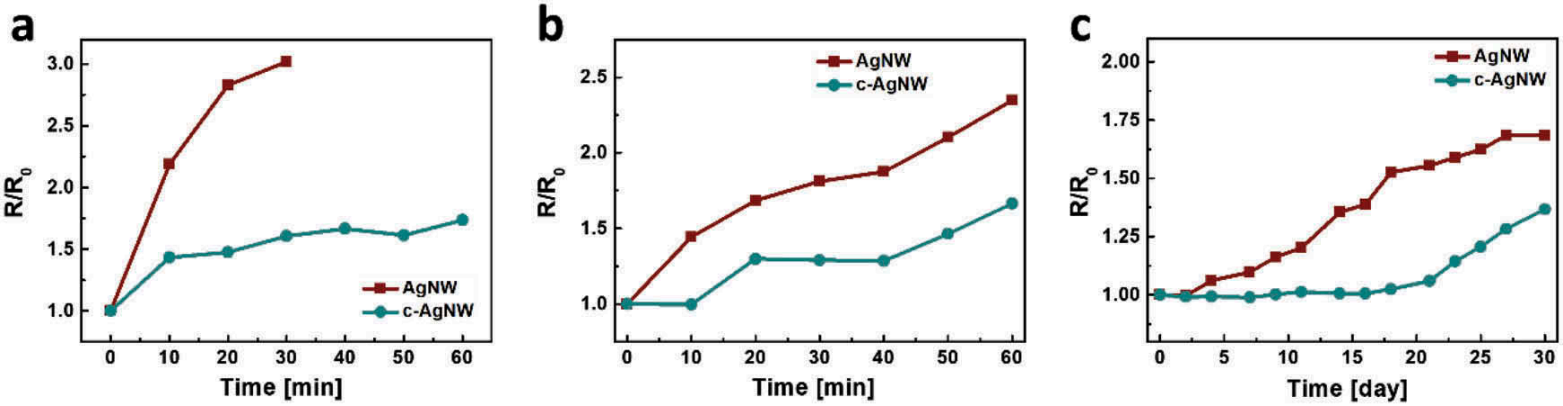
Changes of the normalized resistance for AgNW and c-AgNWs which are (a) dipped in ethanol, (b) dipped in deionized water, and (c) exposed to air ambient. The c-AgNWs show the significantly improved chemical stability attributed to the protection effect of overcoated PEDOT:PSS.

We investigate chemical stabilities of AgNWs and c-AgNWs, both treated with 11-AA (0.14 wt.%), against various conditions. Figure 5(a) shows the change in resistance of the electrodes dipped in ethanol. The resistance of the AgNW electrode dipped in ethanol is increased by 3.0-fold during dipping for 30 min after that the AgNW networks are peeled-off from the supporting substrate. In contrast, c-AgNWs show the significantly improved chemical stability attributed to the protection effect of overcoated PEDOT:PSS. The sheet resistance of c-AgNWs increases by only 1.7-fold during dipping for 60 min. This result suggests that the interconnection between nanowires can be effectively preserved by the thick PEDOT:PSS protection layer against the penetration of chemicals. This enhanced stability is also observed for the films dipped in deionized water and the films stayed in air ambient (Figure 5(b,c)). The resistances of c-AgNWs are only increased by 1.7- and 1.4-folds, while those of AgNWs are increased by 2.3- and 1.7-folds in deionized water (60 min) and air conditions (30 days), respectively. The greatly enhanced stability of c-AgNWs is useful for highly stable electrodes in wearable electronics.

**Figure 6.**
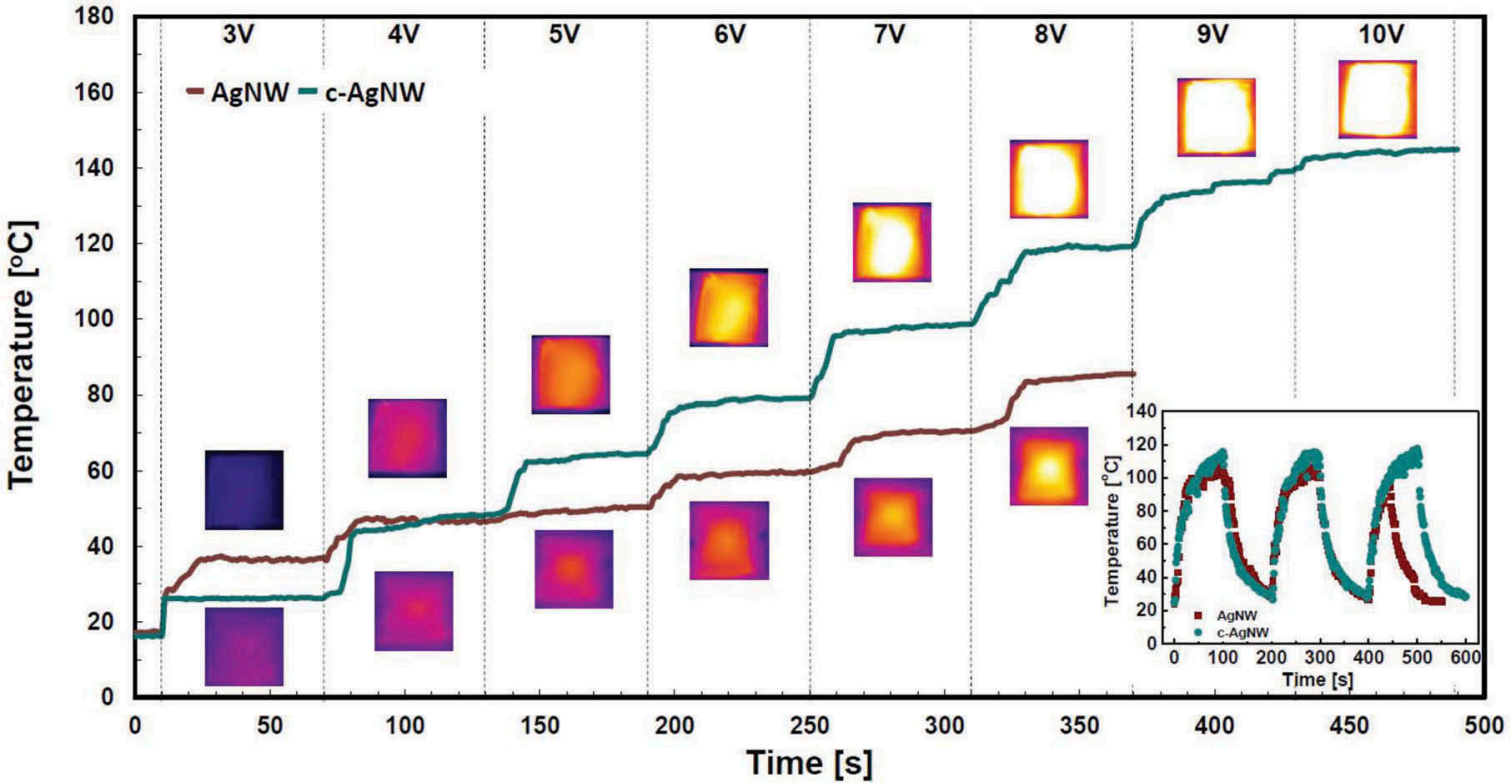
Temperature changes of STHs based on AgNW and c-AgNW as a function of time with increased applied voltage. Inset shows dynamic temperature control of the STHs with AgNW and c-AgNW. The STHs with c-AgNW exhibit more enhanced and stable heating properties in comparison with the STHs with AgNW.

The stretchable transparent heaters (STHs) are realized by AgNW and c-AgNWs with sheet resistances of 26 and 20 ohm/sq, respectively. All films for STHs are chemically treated with 11-AA (0.14 wt.%). Figure 6 presents the Joule heating characteristics of the STHs, where a DC voltage is applied to the STHs and is increased by every 60 s from 3 to 10 V. The produced temperature clearly depends on the applied voltage. The STH with c-AgNWs generates the much higher temperature, reaching 140 °C, compared to the AgNW-based STH at given voltages. Above 8 V, the failure of AgNW-based STH is observed, while STHs with c-AgNWs work until a high voltage of 10 V. These results indicate that the overcoated PEDOT:PSS layer in c-AgNW-based STHs enhances the formation of conducting pathways between nanowires and prevents from the failure of wire-to-wire contact at high temperatures. The failure mode of devices at high voltages is caused by a damage of nanowire junctions induced by high temperature. The operational stability of STHs under a constant bias voltage of 5 V is shown in the inset of Figure 6. STHs with c-AgNW exhibit more enhanced and stable heating properties in comparison with STHs with AgNW during the repetitive on-off cycles, indicating that the overcoated PEDOT:PSS effectively suppresses the damage of the wires under repeated thermal stress.

**Figure 7.**
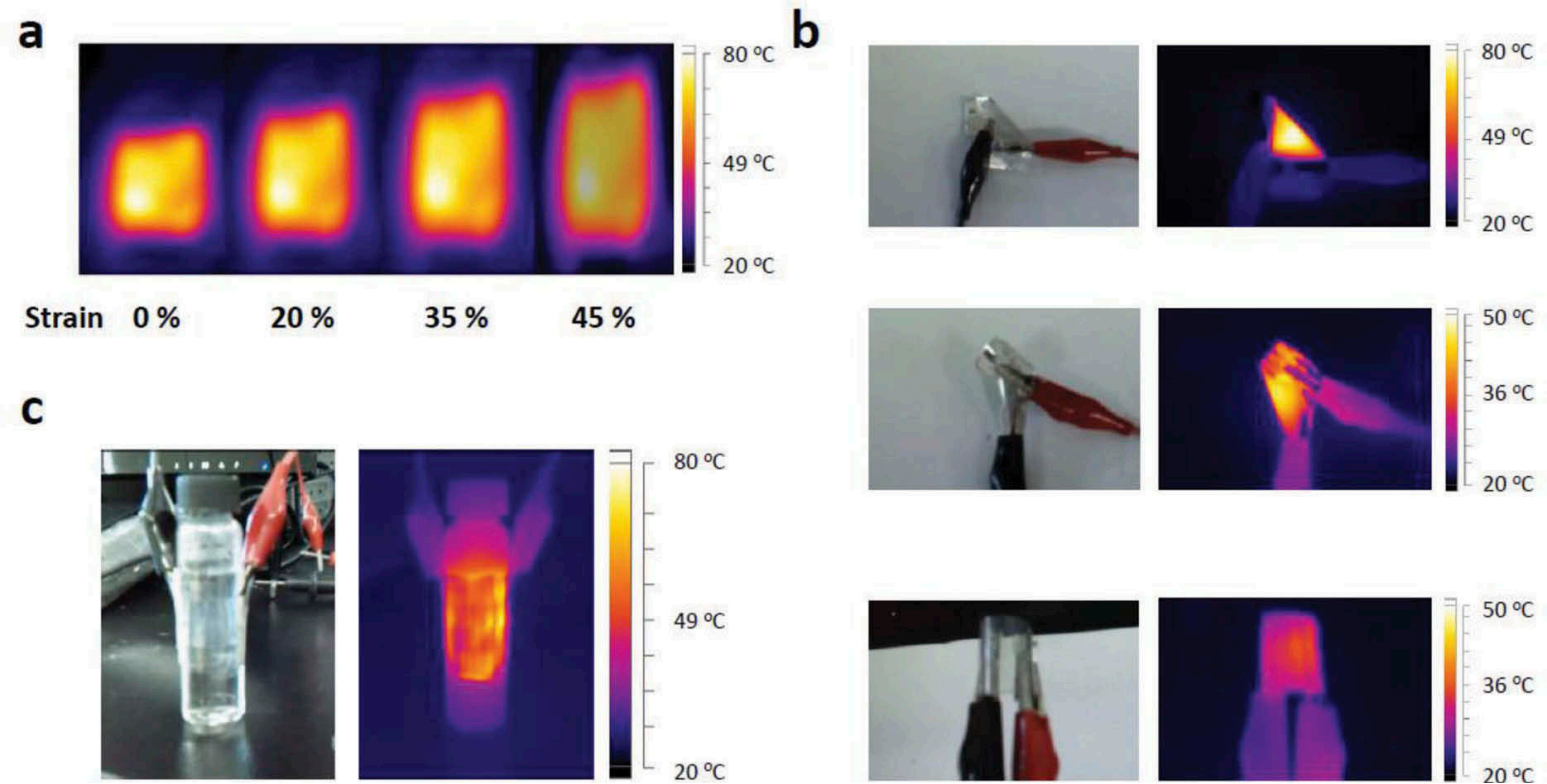
(a) IR image of the STH with c-AgNW under various tensile strains. (b) Photographs and IR images of STHs based on c-AgNW under twisting and bending conditions. (c) Photograph and IR image of STH with c-AgNW attached on a vial. The STHs are easily twisted and attached on a bent surface.

Figure 7(a) shows the IR images of the STHs based on c-AgNW treated with 0.14 wt.% 11-AA under various tensile strains at a constant voltage. The STHs with c-AgNWs stretched up to 45% exhibit the outstanding stretchability and the good temperature distribution on the films. The temperature difference does not significantly differ for all films applied with various strains (Supplementary Material Fig. S3). Figure 7(b,c) shows the superb elasticity of STHs under various twisting and bending conditions. The STHs are easily twisted and attached on a bent surface, showing good conformal performance with various form factors. In addition, we integrate c-AgNWs as a stretchable transparent electrode to the stretchable alternating current electroluminescence device (Supplementary Material Fig. S4), presenting a promising performance for applications in displays and lighting.

## 4. Conclusions

The electrical, optical, and mechanical properties of AgNW films are simultaneously enhanced by the introduction 11-AA, forming a strong chemical bond between nanowires and PDMS. The transparent electrodes treated with 11-AA exhibit a low sheet resistance of 26.0 ohm/sq and a high transmittance of 90%. The resistance change of the stretchable AgNW/PEDOT:PSS thin-films is only about 10% when the film is stretched by 120%. In addition, the embedment of AgNW into the conductive PEDOT:PSS film provides the remarkably improved chemical stability as the overcoated PEDOT:PSS effectively protects chemical penetration in the films. The STHs prepared with c-AgNWs exhibit much enhanced and stabilized Joule heating performances. The stretchable devices exhibit good elastic behaviors, which can be stretched, bent, and twisted. We believe that the surface modification strategy investigated here has high availability and can be a key step toward future wearable applications.
