# Supplementary material for: Simultaneously enhanced optical, electrical, and mechanical properties of highly stretchable transparent silver nanowire electrodes using organic surface modifier
Source: Sci Technol Adv Mater. 2019 Feb 18;20(1):116–23. doi: 10.1080/14686996.2019.1568750 (PMC6383608; doi:10.1080/14686996.2019.1568750)
Supplement: Supplemental Material [file TSTA_A_1568750_SM3321.docx]

**Supplementary Material**

**Simultaneously enhanced optical, electrical, and mechanical properties of highly stretchable transparent silver nanowire electrodes using organic surface modifier**

*Siti Aisyah Nurmaulia Entifar^1^, Joo Won Han^1^, Dong Jin Lee^1^, Zeno Rizqi Ramadhan^1^, Ju Hee Hong^1^, Changhun Yun^2^, and Yong Hyun Kim^1,*^*

^1^Department of Display Engineering, Pukyong National University, Busan 48513, Republic of Korea

^2^Center for Nano-Photonics Convergence Technology, Korea Institute of Industrial Technology (KITECH), Gwangju 61012, Republic of Korea

^*^Corresponding author.

E-mail: yhkim113@pknu.ac.kr

Tel. : +82-51-629-6418; Fax : +82-51-629-6408


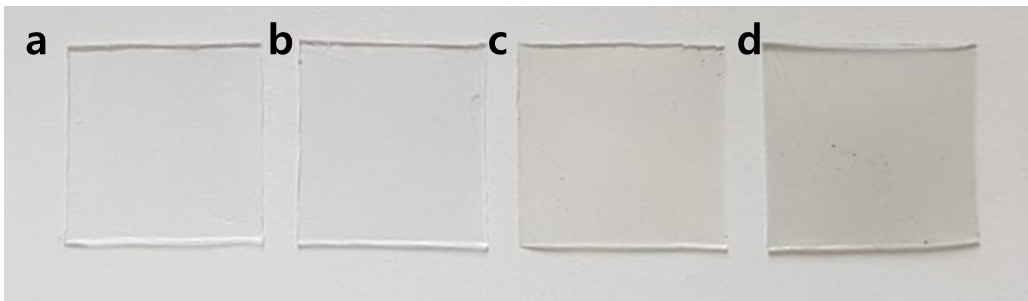


**Figure S1.** Photographs of (a) the PDMS substrate, (b) the PDMS substrate with 11-AA treatment (0.14 wt.%), (c) AgNWs on the PDMS substrate with 11-AA treatment (0.14 wt.%), and (d) c-AgNWs on the PDMS substrate with 11-AA treatment (0.14 wt.%).

**Figure S2.** Transmittance and sheet resistance of the films. The transmittance of AgNW/PEDOT:PSS composite film is higher than that of PEDOT:PSS film, which might be due to the different thickness of coated PEDOT:PSS layers on the different underlying films.


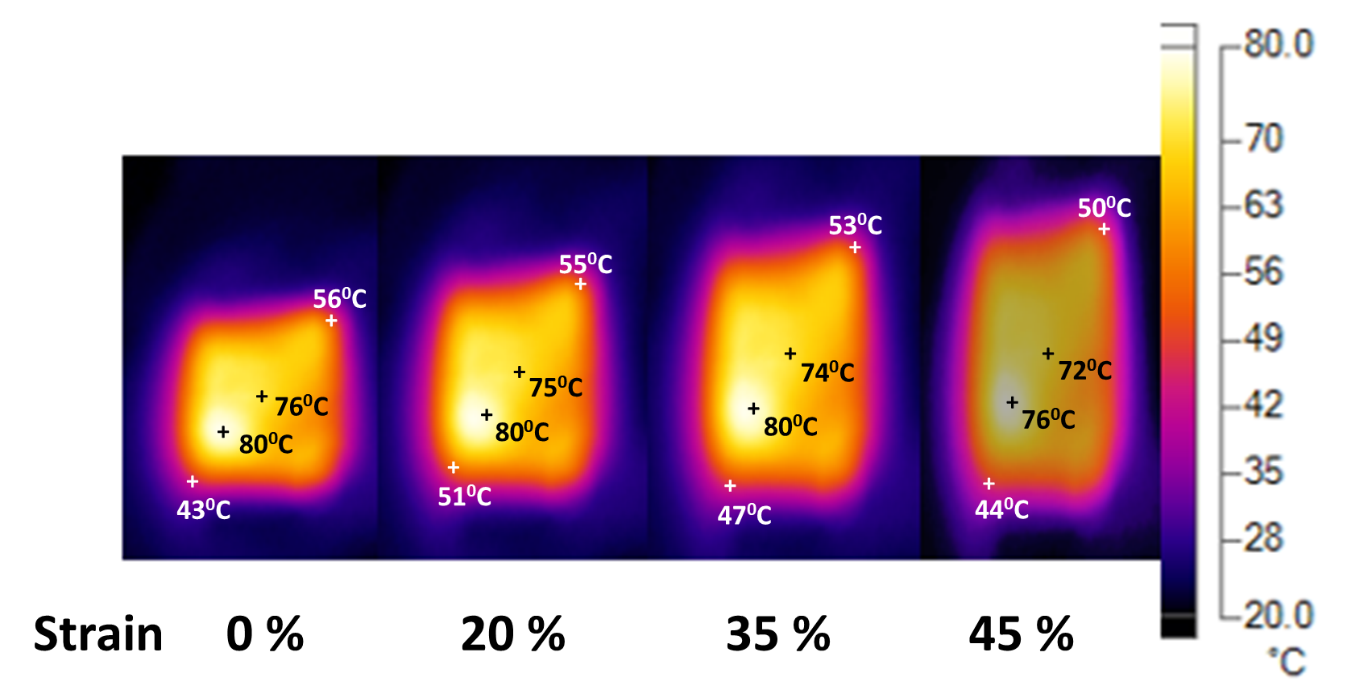


**Figure S3**. (a) Temperature distributions of the STH with c-AgNW under various tensile strains.


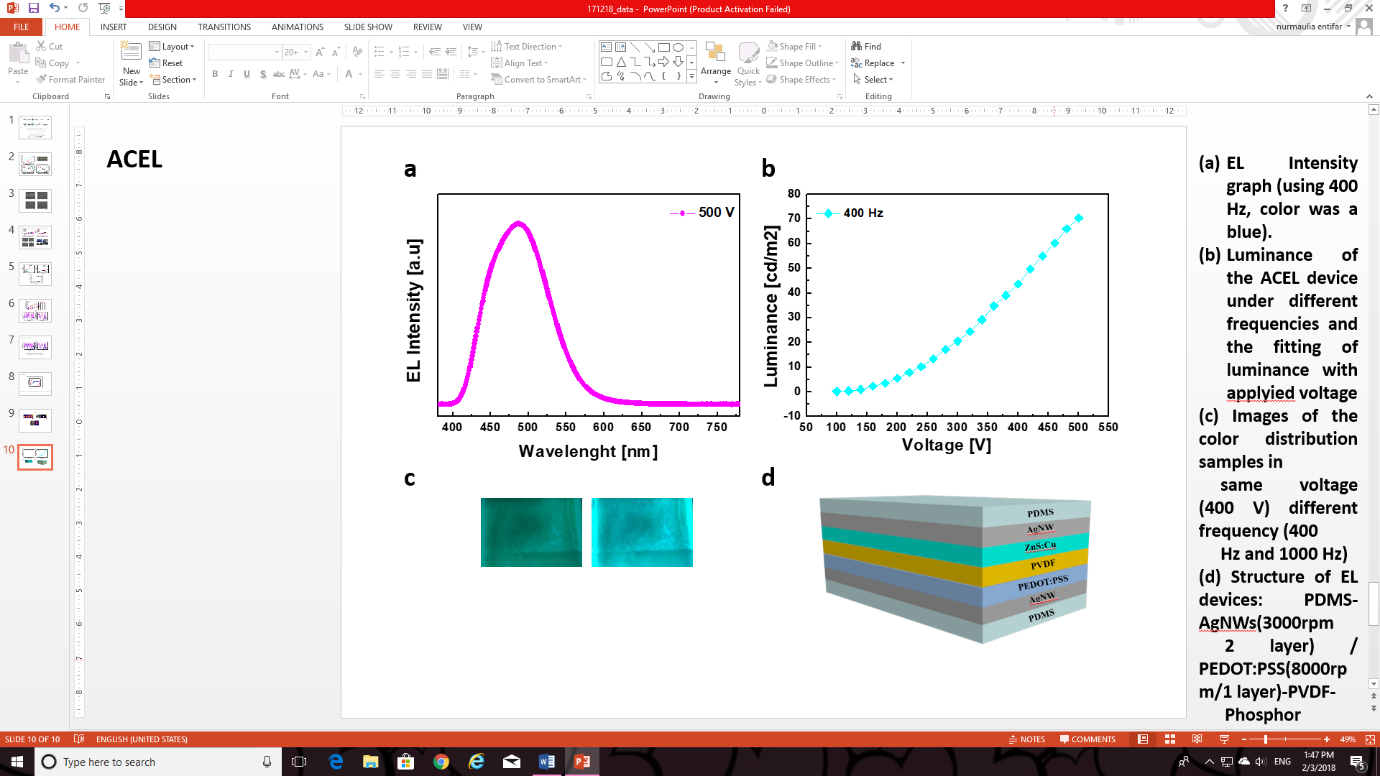


**Figure S4**. (a) Electroluminescent intensity (at 500 V) and (b) luminance (at 400 Hz) of the stretchable alternating current electroluminescence device. (c) Photographs of stretchable alternating current electroluminescence device with a frequency 400 Hz (left) and 1000 Hz (right) and a voltage of 400 V. (d) Schematic structure of stretchable alternating current electroluminescence device.


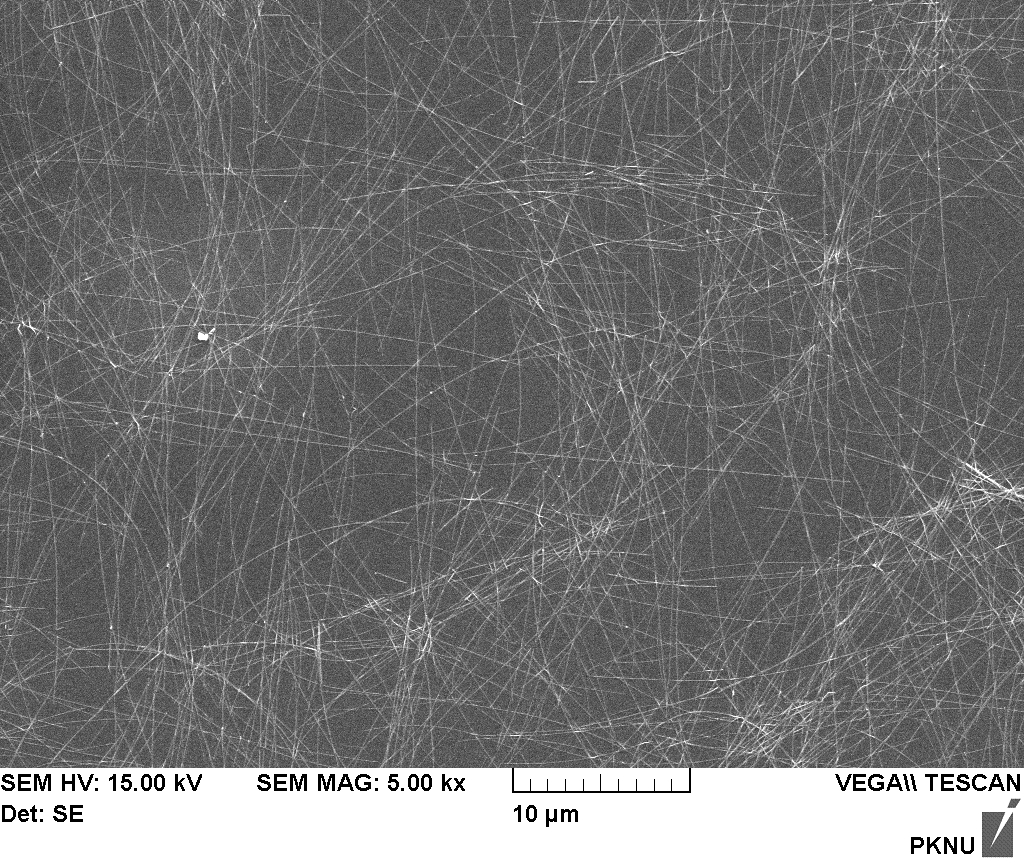


**Figure S5**. The SEM image of untreated AgNWs on the PDMS film. The average length and diameter of the AgNWs were about 25 μm and 32 nm, respectively.
